# Retrospective analysis of the surgical management of spontaneous supratentorial intracerebral hemorrhage: A single-center study

**DOI:** 10.5339/qmj.2021.53

**Published:** 2021-10-18

**Authors:** Ahmed Shaaban, Maher Saqqur, Ahmed Saleh, Alaaeldin Ahmed, Hussain Hussain, Arun Babu R, Abdulnasser Alyafai, Sirajeddin Belkhair, Ali Ayyad

**Affiliations:** ^1^Department of Neurosurgery, Hamad Medical Corporation, Doha, Qatar. E-mail: ashaaban4@hamad.qa; ^2^Department of Medicine Division of Neurology, University of Alberta, Edmonton, Alberta, Canada.; ^3^Department of Neurosurgery, Weill Cornell Medical College, Doha, Qatar.; ^4^Department of Surgery, Michigan State University, Lansing, USA.; ^5^Department of Neurosurgery, Saarland University hospital, Homburg, Germany.; ^6^Trillium Hospital University of Toronto Mississauga, Ontario, Canada.

**Keywords:** spontaneous supratentorial intracerebral hemorrhage, craniotomy hematoma evacuation, functional outcome intracerebral hematoma

## Abstract

Background: Intracerebral hemorrhage (ICH) remains a devastating disease with high morbidity and mortality. The mortality rate ranges from 40% at 1 month to 54% at 1 year, and only 12%–39% achieve good outcomes and functional independence. The current management guidelines for spontaneous supratentorial ICH are still controversial.

Objective: Patients who presented with ICH and underwent craniotomy with hematoma evacuation or minimal procedures from January 2016 to May 2020 were included in the analysis. Several clinical, radiological, and surgical variables were collected to identify the variables most likely related to lower mortality and better functional outcomes.

Results: A total of 87 patients presented with HMC with ICH from January 2016 to May 2020.

The mean age was 44.7 (42.2–47.2) years. There were 76 male (87.4%)/11 female (12.6%) patients, which reflect the population distribution in Qatar, which is mainly male predominant. Although Qatar is mainly a Middle-Eastern country, the ethnic distribution of patients was mainly of South Asian and Indian (60.9%) and Far-Eastern (20.7%) ethnicities because of diversity. The mean baseline Glasgow coma scale (GCS) was 8.2+/ − 3.7. The mean baseline functional independence measure (FIM) score was 59.4+/ − 36.7. Most hematomas were located in the basal ganglia (79.3%%). Baseline characteristics based on long-term outcomes are summarized in Table 1. The following variables were correlated with poor outcome: low GCS on postoperative day 1 (P = 0.06), low FIM score (P = 0.006), ICH location (P = 0.04), distance of the closest point to the surface (P = 0.009), and presence of uncal herniation (P = 0.04). The baseline characteristics based on mortality are outlined in Table 2. The following variables were correlated with mortality: diabetes mellitus (P = 0.02), baseline GCS (P = 0.04), GCS on postoperative day 1 (P = 0.002), unequal pupils (P = 0.05), and postoperative midline shift (P = 0.001).

Conclusion: The preoperative clinical neurological status as well as mass effect (uncal herniation and midline shift) can be determinants of functional outcome and mortality. A deeper hematoma may result in poor functional outcome because of more surgical damage in functional brain tissues. Thus, the goal of surgery in spontaneous supratentorial ICH is to reduce mortality, but no evidence support that it can improve functional outcome. Although our findings are interesting, more prospective studies with a larger sample size are needed to confirm our results.

## Introduction

Intracerebral hemorrhage (ICH) remains a devastating disease with major morbidity and mortality^1^. The mortality rate ranges from 40% at 1 month to 54% at 1 year, and only 12%–39% achieve good outcomes and functional independence^2^.

The current management guidelines for spontaneous supratentorial ICH are still controversial. The condition represents a challenge for neurosurgeons and neurologists, as it is an emergency, and decisions about the best management for the patient should be fast and effective^1^. The surgical management options include open surgery (craniotomy with hematoma evacuation plus or minus decompression or decompression only) or minimally invasive surgery (MIS)^3^.

Several trials have examined the role of surgery in ICH. The main trials are the STICH-I and STICH-II, which compared early surgery with conservative management in patients with spontaneous supratentorial ICH ^4, 5^. STICH-I found no difference between the two groups regarding mortality and functional outcome, unlike STICH-II, which suggested that early surgery may have a survival advantage if hemorrhage is superficial and no intraventricular hemorrhage (IVH) is present^1, 4-6^. Recently, some trials have compared medical and open surgical methods with minimally invasive techniques (MISTIE-I, MISTIE-II, MISTIE-III, CLEAR, and SLEUTH)^7-11^. The peroxisome proliferator-activated receptor gamma agonist pioglitazone has been evaluated in some MIS trials; theoretically, it enhances phagocytosis and improves oxidative stress and inflammation^12^. Therefore, based on the available literature, we are on the path but still have not reached our objective of establishing guidelines for the best management of spontaneous supratentorial ICH to provide the best care of our patients and thus achieve low mortality and better functional outcomes.

Our state has a multicultural demography in its population, making it an excellent region to study the etiology and mechanism of ICH. In Qatar, 84% of the population are male because of the large majority of expatriates, and 82% are < 65 years old. In addition, the entire state is covered by a major tertiary hospital that receives most of the stroke cases in the country, allowing for a population-based data source.

Thus, this study aimed to analyze and identify clinical, radiological, surgical variables probably associated with less mortality and better functional outcome by comparing our results with those of previous studies. We hope that the study findings can guide further research and help establish clear guidelines for the management of spontaneous supratentorial ICH.

## Methods

This study was a retrospective collection of the data of patients who presented with ICH to our hospital between January 2016 and May 2020. This study was approved by the local institutional review board and medical research committee.

Patients were included in the study if they fulfilled the following criteria: 1) diagnosis of spontaneous supratentorial ICH in patients who underwent craniotomy with hematoma evacuation plus or minus decompression or patients who underwent decompression alone; 2) volume > 30 ml or midline shift (MLS) > 5 mm; and 3) no identifiable cause of ICH other than hypertension based on computed tomography (CT) angiogram or magnetic resonance imaging.

Exclusion criteria

Patients with ICH who had a defined etiology of their ICH besides ICH (vascular lesion [aneurysm], venous sinus thrombosis, brain tumor, traumatic ICH, intracranial infection, and hemorrhagic infarction) were excluded from the study.

The following data were collected from the electronic charts of the patient population: patient age, sex (male or female), and ethnicity (South Indian, Middle Eastern, North African, local, White, African, Far-eastern).

## Clinical variables

Diabetes mellitus (DM), hypertension, cardiac disease, smoking history, anticoagulation, preoperative and postoperative day 1 Glasgow coma scale (GCS), preoperative pupil status (equal vs. nonequal; reactive vs nonreactive), and postoperative day 1 status of the pupils (dilated fixed, equal reactive, anisocoria, and equal nonreactive) were evaluated.

ICH score (from 0 to 6) was evaluated based on five factors: GCS (0 = 13–15, 1 = 5–12, 2 = 3–4), age (0 =  below 80, 1 =  above 80), IVH (0 =  absent, 1 =  present), supratentorial origin = 0 vs infratentorial hemorrhage = 1, and ICH volume (below 30 cc = 0 vs above 30 cc = 1).

## Radiological variables

Patients’ plain head CT scans were analyzed preoperatively and postoperatively to identify the following data: location (basal ganglia, cortical, or thalamus); involved hemisphere (right vs left); ICH volume (cm^3^) measured using the method with the largest length in three dimensions divided by two (equation abc/2); preoperative and postoperative distance of the closest point of hematoma to the brain surface (cm), MLS (mm) measured at the level of the septum pellucidum from a midline drawn at the level of the attachments of the falx cerebri anterior and posterior to inner skull tables; preoperative and postoperative edema (mild, moderate, or severe); brain stem involvement or noninvolvement; presence or absence of IVH preoperatively and postoperatively; presence or absence of hydrocephalus preoperatively and postoperatively; uncal herniation; and residual volume postoperative (no residual, near-total evacuation, partial evacuation, or no evacuation only decompression).

## Surgical variables

The following surgical data were collected: type of surgery (craniotomy with hematoma evacuation, craniotomy with hematoma evacuation + decompression, or decompression alone), external ventricular drain was inserted or not inserted, intracranial pressure (ICP) monitoring was performed or not performed, brain navigation was included or not included during surgery, time from initial plain CT of the head to surgery (hours), and adverse events (redo surgery, infection, or none).

## Clinical outcomes

**Good long-term outcomes were defined in this study: mRS of 0–2 at 3 months. The mortality rate was measured at 30 and 90 days.**

Functional independence measure (FIMs) is a widely accepted functional assessment measure that is used during inpatient rehabilitation. The FIM is an 18-item ordinal scale that is used in all diagnoses within a rehabilitation population. FIM scores range from 1 to 7 (1 =  total assistance; 7 =  complete independence). Scores <  6 require the help of another person for supervision or assistance. The FIM assesses independent performance in self-care, sphincter control, transfer, locomotion, communication, and social cognition. By adding the points for each item, the possible total scores for the level of independence range from 18 (lowest) to 126 (highest) points.

## Statistical analysis

By using SPSS for statistical analysis, data were coded with numbers (e.g., 0, 1, 2, 3…). Data were classified into either categorical or continuous numerical variables. Dependent variables were 30-day mortality and modified Rankin scale (mRS) (further classified as good outcome [0–2] or poor outcome [3–6]). All other independent variables were related to 30-day mortality and mRS. The chi-square test was used for categorical variables, and the one-way analysis of variance was used for continuous numerical variables.

Logistic regression analysis was performed to measure the odds ratio (ORs) of mortality and poor outcome, with adjustment for common risk factors, and presented as OR with confidence interval 95%. Continuous data were checked for normality using means ±  standard deviation. P value <  0.05 was considered significant.

## Results

A total of 87 patients presented with HMC with ICH from January 2016 to May 2020. The mean age was 44.7 (42.2–47.2) years. There were 76 male (87.4%) and 11 female (12.6%) patients, which reflects the population distribution in Qatar, which is mainly male predominant. Although Qatar is mainly a Middle-Eastern country, the ethnic distribution of patients was mainly of South Asian and Indian (60.9%) and Far-Eastern (20.7%) ethnicities because of diversity. The mean baseline GCS was 8.2+/ −  3.7. The mean baseline FIM was 59.4+/ − 36.7. Most hematomas were located in the basal ganglia (79.3%).

The procedures performed were craniotomy + hematoma evacuation (78.2%), craniotomy + hematoma evacuation + decompression (13.8%), and decompression alone (8.0%). The mortality, survival, good outcomes (3-month mRS <  3 points), and poor outcome rates (mRS 3–6 points) were 8% (n = 7), 92% (n = 80), 11.5% (n = 10), and 88.5% (n = 77), respectively.

Baseline characteristics based on long-term outcomes are summarized in Table 1. The following variables were correlated with poor outcome: low GCS on postoperative day 1 (P = 0.06), low FIM (P = 0.006), ICH location ICH (P = 0.04), distance of the closest point to the surface (P = 0.009), and presence of uncal herniation (P = 0.04) (Table 1).

The baseline characteristics based on mortality are outlined in Table 2. The following variables were correlated with mortality: DM (P = 0.02), baseline GCS (P = 0.04), GCS on postoperative day 1 (P = 0.002), unequal pupils (P = 0.05), and postoperative MLS (P = 0.001) (Table 2).

In the multiple logistic regression analysis with poor outcome as the dependent variable, uncal herniation (P = 0.06; adjusted OR, P = 0.07) and location in the basal ganglia (P = 0.02; adjusted OR, P = 0.5) were significantly correlated with poor outcomes (Table 3).

In the multiple logistic regression analysis with mortality as the dependent variable, DM (P = 0.021; adjusted OR, P = 0.02), and equal vs unequal preoperative pupils (P = 0.04; adjusted odds ratio, P = 0.9) were significantly correlated with mortality (Table 4).

## Discussion

This study showed that baseline clinical radiological and surgical findings can help predict clinical outcomes in patients with ICH. The presence of neuroimaging findings of ICH location and uncal herniation correlated with poor outcomes. However, the clinical status, especially GCS on postoperative day 1, of the patient was more related to both outcome and mortality. Our finding that the distance of the closest point to the surface in craniotomy correlated with outcome is an interesting observation.

In this study, we found that the deeper the hematoma, the worse the surgical outcome. This could advocate for minimally invasive procedures as the ideal method of surgical treatment, which is the prevailing concern in current randomized controlled trials.

Decisions regarding surgical intervention in spontaneous supratentorial ICH are still challenging among neurosurgeons in day-to-day practice. On patient evaluation, the most significant epidemiological, clinical, radiological, and surgical factors that can predict the overall mortality and functional outcome of every patient are still unknown. In our study, we tried to evaluate our experience and analyze as many factors as possible to identify the trends and possible associations between different factors and outcomes.

In a previous study, Kim et al. compared patients who underwent craniotomy with hematoma evacuation and patients who underwent decompressive craniectomy in addition to hematoma evacuation. Factors associated with less 30-day mortality were preoperative GCS > 9, hematoma volume <  80, and time from ictus to surgery, while predictors of better functional outcome were age, IVH, time from ictus to surgery, and postoperative MLS. In contrast to our study's findings, postoperative MLS was more related to 30-day mortality^13^.

Different theories can explain the finding that MLS on postoperative day 1 had a significant association with mortality (the more severe the MLS on postoperative day 1, the more likely the patient will die). First, hematoma evacuation will reduce all factors mentioned previously that are related to cytotoxic brain edema. Second, clotted hematoma has less mass effects and consequently low ICP^14, 15^.

The distance from the closest point to the surface was 5.7 times more likely to be associated with a poor outcome (P = 0.009), which is an interesting finding. This means that the closer the hematoma to the surface, the better the functional outcome. According to the STICH-II trial, early surgery can have a small clinical survival benefit for superficial hematoma without IVH, but no evidence can support the improvement in functional outcomes^5^.

Based on the trend that superficial hematoma is associated with better functional outcomes, we can suggest that the less normal brain tissue damage, the better the postoperative functional outcome and better prognosis. By translating that theory into the clinical practice, we can advocate for minimally invasive ICH evacuation, especially for deep-seated hematoma, to protect normal brain tissue as much as possible, in contrast to open surgery where dissection and retraction can cause significant brain damage^3^.

Recently, many trials, including MISTIE-II and MISTIE-III, have compared minimally invasive hematoma evacuation with conservative management. The MISTIE technique aimed to decrease the hematoma size to <  15 ml: a rigid cannula was inserted through a burr hole, and clot aspiration was performed with a 10-ml syringe until there was resistance. Thereafter, the cannula was replaced by a catheter under imaging guidance into the hematoma. The placement was confirmed by CT at 6 h after intrathecal administration of alteplase 1 mg in 1 ml of saline every 8 h, followed each time by a 3-ml saline flush and closure of the catheter for 1 h. The goal was to achieve a hematoma volume <  15 ml or to administer nine doses of alteplase, whichever came first. Although MISTIE proved to be a safe technique, it failed to provide better functional outcomes than conservative management^3, 7, 8^. Some trials are ongoing, including SCUBA (stereotactic ICH underwater blood aspiration, ENRICH trial (early minimally invasive removal of ICH), INVEST trial (minimally invasive endoscopic surgical treatment with Apollo/Artemis in patients with brain hemorrhage), and MIND trial. We hope that they can show promising results toward ICH management more in the direction of MIS ^16, 17^.

Postoperatively, DM and MLS were the two factors most significantly related to 30-day mortality. According to 2015 guidelines from the American Stroke Association, hyperglycemia and hypoglycemia should be avoided (Level C evidence: Class 1)^1^. Two previous studies have concluded that hyperglycemia on admission, even in patients with no DM history, increased the risk of mortality and poor functional outcome^18, 19^. Based on our findings, patients with DM were 12.972 times more likely to die within 30 days than patients without DM.

GCS on postoperative day 1 and preoperative equal or unequal pupils were two factors related to 30-day mortality. The same was true for uncal herniation and location in the basal ganglia in relation to functional outcome. However, these trends can be investigated by additional large studies.

The type of surgery did not show any difference, but interestingly, no deaths were recorded in patients who had decompression + hematoma evacuation or patients who underwent decompression alone.

In a review by Mendelow^20^, a meta-analysis of three STITCH trials (STITCH I, STITCH II, STITCH Trauma) showed that patients treated with craniotomy with initial GCS 9-12 may benefit from surgery, especially with early intervention.

Gregson et al. reviewed and performed a meta-analysis of trials conducted for surgical management of ICH until 2010 (14 trials included). They suggested that better outcomes can be achieved with early surgery within 8 h, hematoma 20–50 ml, GCS ≥ 9, and age 50–69 years^21^.

**This study has several limitations.** First, this study has a retrospective design and prone to the effects of confounders. Second, as it is a single-center study, the results cannot be generalized. Third, the sample size was small; a larger sample with a multicenter registry is needed to confirm our findings. Fourth, the sample size was not enough to study all potential factors or variables. Owing to the mentioned limitations, our study's findings can only be used for hypothesis generation rather than drawing firm conclusions.

**In conclusion,** clinical neurological status preoperative as well as mass effect (uncal herniation and MLS) can be determinants of functional outcome and mortality. In addition, a deeper hematoma may result in poor functional outcome because of more surgical damage in functional brain tissues. The goal of surgery in spontaneous supratentorial ICH is to reduce mortality, but no evidence supports it can improve functional outcome. Although our findings are interesting, more prospective studies with a larger sample size are needed to confirm our results.

## Figures and Tables

**Table 1:** Baseline characteristics based on long-term outcomes

Variables	Good outcomes (mRS 0–2)	Poor outcomes (mRS 3–6)	P value

Total	11.5% (N = 10)	88.5%(N = 77)	1

Age	39.9 (28.4–51.4) (N = 10)	45.3(42.8–47.8) (N = 77)	0.2

Sex			

Male/ Female	11.8%(N = 9)/ 9.1%(N = 1)	88.2%(N = 67)/ 90.9%(N = 10)	1

Ethnicity			

Unknown	100%(N = 1)	0	0.001

South Asian, Indian	13.2%(N = 7)	86.8%(N = 46)	0.001

MENA	0	100%(N = 2)	0.001

Qatari	0	100%(N = 10)	0.001

White	100%(N = 1)	0	0.001

African	50%(N = 1)	50%(N = 1)	0.001

Far-Eastern	0	100%(N = 18)	0.001

DM			

Diabetic	22.2%(N = 4)	77.8%(N = 14)	0.2

HTN			

HTN	11.7%(N = 7)	88.3%(N = 53)	0.8

Cardiac disease	0	6	

Cardiac	0	100%(N = 6)	0.3

Smoking			

Smoker	0	100%(N = 1)	0.8

GCS preop	9.2(6.2–12.2)(N = 10)	8.03(7.2–8.9)(N = 77)	0.4

GCS on postop day 1	10.30(7.7–12.9)(N = 10)	7.83(6.9–8.7)(N = 77)	0.06

Pupils preop reactive vs non			

reactive	12%(N = 10)	88.0%(N = 73)	1

Unreactive on line only	0.00%	100.0%(N = 4)	1

Pupils preop equal vs non			

equal	11.9%(N = 8)	88.1%(N = 59)	1

Unequal one line only	10%(N = 2)	90.0%(N = 18)	1

Pupils postop			

Equal reactive	13.2%(N = 10)	86.8%(N = 66)	0.4

Anisocoric	0	100%(N = 11)	0.4

ICH score			

0	33.3%(N = 1)	66.7%(N = 2)	0.2

1	14.3%(N = 2)	85.7%(N = 12)	0.2

2	17.6%(N = 6)	82.4%(N = 28)	0.2

3	3.4%(N = 1)	96.6%(N = 28)	0.2

4	0	100%(N = 7)	0.2

FIM total score	90.11(67.5–112.7)(N = 9)	54.9(47–63.9)(N = 63)	0.006

hemisphere			

Right/Left	13%(N = 6)/ 9.8%(N = 4)	87%(N = 40)/ 90.2%(N = 37)	0.7

Location			

Basal ganglia	7.2%(N = 5)	92.8%(N = 64)	0.04

Thalamus	0.00%	100%(N = 1)	0.04

Cortical	29.4% (N = 5)	70.6% (N = 12)	0.04

Volume, ml	57.9(20.1–95.8)(N = 10)	59.9(52.6–67.2)(N = 77)	0.9

MLS, mm	7.3(6.4–8.3)(N = 10)	7.2(6.4–7.9)(N = 77)	0.9

Distance of the closest point to the surface, cm	0.4(-.02 to (-.8)(N = 10)	.9(.8–1)(N = 77)	0.009

IVH			

Absent	13.5%(N = 7)	86.5%(N = 45)	0.7

Present	8.6%(N = 3)	91.4%(N = 32)	0.7

Edema			

Mild	11.8%(N = 4)	88.2%(N = 30)	0.9

Moderate	9.5%(N = 2)	90.5%(N = 19)	0.9

Severe	12.5%(N = 4)	87.5%(N = 28)	0.9

Brain stem			

Present	0	100%(N = 2)	1

Hydrocephalus			

Present	0	100%(N = 5)	1

Uncal herniation			

Present	18%(N = 9)	82%(N = 41)	0.04

MLS postop	3.4(2.1–4.6)(N = 10)	3.7(2.9–4.5)(N = 76)	0.8

Residual volume			

No residual	27.8%(N = 5)	72.2%(N = 13)	0.1

Near-total evacuation	6.9%(N = 2)	93.1%(N = 27)	0.1

partial evacuation	9.7%(N = 3)	90.3%(N = 28)	0.1

no evacuation only decompression	0	100%(N = 8)	0.1

Edema postop			

Mild	18.2%(N = 6)	81.8%(N = 27)	0.3

Moderate	10% (N = 2)	90.0%(N = 18)	0.3

Severe	6.1%(N = 2)	93.9%(N = 31)	0.3

IVH postop			

Absent	14.9%(N = 7)	85.1%(N = 40)	0.3

Present	7.7%(N = 3)	92.3%(N = 36)	0.3

Hydrocephalus postop			

Absent	12%(N = 10)	88%(N = 73)	1

Present	0	100%(N = 3)	1

Time from initial CT to surgery	8.1(-3.6–19.8)(N = 9)	13.6(5.9–21.2)(N = 77)	0.6

Type of surgery			

Craniotomy hematoma evacuation	13.2%(N = 9)	86.8%(N = 59)	0.5

Craniotomy hematoma evacuation plus decompression	8.3%(N = 1)	91.7%(N = 11)	0.5

Decompression only	0	100%(N = 7)	0.5

ICP			

Present	20%(N = 1)	80%(N = 4)	0.5

EVD			

present	13.3%(N = 2)	86.7%(N = 13)	0.6

Brain Navigation			

present	6.5%(N = 2)	93.5%(N = 29)	0.5


Categorical variables are presented as a percentage % within each variable, while continuous data are presented as means with 95% confidence interval

N, numbers; CT, computed tomography; IVH, intraventricular hemorrhage; ICP, intracranial pressure; EVD; MLS, midline shift

**Table 2:** Baseline characteristics based on mortality

Variables	No mortality	Mortality	P value

Total	92%(N = 80)	8%(N = 7)	1

Age	44.9 (42.3–47.7)(N = 80)	41.1(32.6–49.7)(N = 7)	0.4

Sex			

Male	92.1%(N = 70)	7.9%(N = 6)	1

Female	90.9%(N = 10)	9.1%(N = 1)	1

Ethnicity			

Unknown	100%(N = 1)	0	0.8

South Asian, Indian	94.3%(N = 50)	5.7%(N = 3)	0.8

Middle-eastern, North Africa	100%(N = 2)	0	0.8

Qatari	80%(N = 8)	20%(N = 2)	0.8

White	100%(N = 1)	0	0.8

African	100%(N = 2)	0	0.8

Far-eastern	88.9%(N = 16)	11.1%(N = 2)	0.8

DM			

Diabetic	88.9%(N = 16)	11.1%(N = 2)	0.02

HTN			

HTN	91.7%(N = 55)	8.3%(N = 5)	0.4

Cardiac disease			

Cardiac	83.3%(N = 5)	16.7%(N = 1)	0.6

Smoking			

Smoker	100%(N = 1)	0	0.2

GCS preop	8.4(7.6–9.2)(N = 80)	5.4(3.4–7.5)(N = 7)	0.04

GCS postop day 1	8.5(7.7–9.3)(N = 80)	3.9(1.4–6.3)(N = 7)	0.002

Pupils preop reactive vs non			

Reactive	92.8%(N = 77)	7.2%(N = 6)	0.3

Unreactive	75.0%(N = 3)	25.0%(N = 1)	0.3

Pupils preop equal vs non			

Equal	95.5%(N = 64)	4.5%(N = 3)	0.05

Unequal	80.0%(N = 16)	20.0%(N = 4)	0.05

Pupils postop			

Equal reactive	93.4%(N = 71)	6.6%(N = 5)	0.2

Anisocoric	81.8%(N = 9)	18.2%(N = 2)	0.2

ICH score			

0	100%(N = 3)	0	0.2

1	100%(N = 14)	0	0.2

2	97.1%(N = 33)	2.9%(N = 1)	0.2

3	82.8%(N = 24)	17.2%(N = 5)	0.2

4	85.7%(N = 6)	14.3%(N = 1)	0.2

FIM total score	65.77(57.68–73.86) (N = 65)	0(N = 7)	0

Hemisphere			

Right	91.3%(N = 42)	8.7%(N = 4)	1

Left	92.7%(N = 38)	7.3%(N = 3)	1

Location			

Basal ganglia	89.9%(N = 62)	10.1%(N = 7)	0.4

Thalamus	100%(N = 1)	0	0.4

Cortical	100% (N = 17)	0	0.4

Volume, ml	581(50.3–65.8)(N = 80)	78.3(49.7–106.9)(N = 7)	0.1

MLS, mm	7.1(6.3–7.7)(N = 80)	9(4.2–13.8)(N = 7)	0.1

Distance of the closest point to the surface, cm	0.9(.7–.9)(N = 80)	1(.6–1.4)(N = 7)	0.6

IVH			

Absent	96.2%(N = 50)	3.8%(N = 2)	0.1

Present	85.7%(N = 30)	14.3%(N = 5)	0.1

Edema			

Mild	91.2%(N = 31)	8.8%(N = 3)	0.9

Moderate	90.5%(N = 19)	9.5%(N = 2)	0.9

Severe	93.8%(N = 30)	6.3%(N = 2)	0.9

Brain stem			

Absent	92.9%(N = 79)	7.1%(N = 6)	0.6

Present	50%(N = 1)	50%(N = 1)	0.6

Hydrocephalus			

Absent	91.5%(N = 75)	8.5%(N = 7)	1

Present	100%(N = 5)	0	1

Uncal herniation			

Absent	97.3%(N = 36)	2.7%(N = 1)	0.2

Present	88%(N = 44)	12%(N = 6)	0.2

MLS postop	3.3518(2.6857–4.0178) (N = 80)	7.7667(2.9619–12.0674) (N = 6)	0.001

Residual volume			

No residual	94.4%(N = 17)	5.6%(N = 1)	0.2

Near-total evacuation	100%(N = 29)	0	0.2

Partial evacuation	87.1%(N = 27)	12.9%(N = 4)	0.2

No evacuation only decompression	87.5%(N = 7)	12.5%(N = 1)	0.2

Edema postop			

Mild	18.2%(N = 6)	81.8% (N = 27)	0.3

Moderate	10.0%(N = 2)	90% (N = 18)	0.3

Severe	6.1%(N = 2)	93.9%(N = 31)	0.3

IVH postop			

Absent	97.9%(N = 46)	2.1%(N = 1)	0.1

Present	87.2%(N = 34)	12.8%(N = 5)	0.1

Hydrocephalus postop			

Absent	94%(N = 78)	6%(N = 5)	0.2

Present	66.7%(N = 2)	33.3%(N = 1)	0.2

Time from the initial CT to surgery	13.854(6.370–21.339) (N = 79)	3.071(1.779–4.364) (N = 7)	0.4

Type of surgery			

Craniotomy hematoma evacuation	89.7%(N = 61)	10.3%(N = 7)	0.4

Craniotomy hematoma evacuation plus decompression	100%(N = 12)	0	0.4

Decompression alone	100%(N = 7)	0	0.4

ICP			

Absent	92.7%(N = 76)	7.3%(N = 6)	0.4

Present	80%(N = 4)	20%(N = 1)	0.4

EVD			

Absent	94.4%(N = 67)	5.6%(N = 4)	0.06

Present	80%(N = 12)	20%(N = 3)	0.06

Brain navigation			

Absent	94.6%(N = 53)	5.4% (N = 3)	0.2

Present	87.1%(N = 27)	12.9% (N = 4)	0.2


Categorical variables are presented as a percentage % within each variable, while continuous data are presented as means with confidence interval 95%

N, numbers; CT, computed tomography; IVH, intraventricular hemorrhage; ICP, intracranial pressure; EVD; MLS, midline shift

**Table 3:** Logistic regression odds ratios and adjusted odds ratios for significant variables according to poor and good outcomes

Variables	Odds ratio (95% CI)	Coefficient (SE)	P value	Adjusted odds ratio (95% CI)	Coefficient (SE)	P value

Uncal herniation	7.9(0.9–65.4)	1.08	0.06	7.6(0.9–66.5)	1.1	0.07

Location 1 basal ganglia	5.3(1.3–21.3)	0.7	0.02	1.9(0.3–10.7)	0.9	0.5


Odds ratio (OR) with 95% confidence interval

**Table 4:** Logistic regression odds ratios and adjusted odds ratios for significant variables according to 30-day mortality

Variables	Odds ratio (95% CI)	Coefficient (SE)	P value	Adjusted odds ratio (95% CI)	Coefficient (SE)	P value

DM	3.5(1.2–10.1)	0.5	0.02	12.9(1.6–102.6)	1.1	0.02

Pupils preop equal vs unequal	5.3(1.1–26.3)	0.8	0.04	1.2(.1–13.1)	1.2	0.9

Pupils preop reactive vs non-reactive	4.3(.4–47.7)	1.2	0.2			


Odds ratio (OR) with 95% confidence interval

GCS on postop day 1, DM, MLS postop, and pupils preop equal vs unequal were adjusted
